# A Case Report of Kleptomania Involved in the Forensic Process

**DOI:** 10.1192/j.eurpsy.2026.11285

**Published:** 2026-07-31

**Authors:** S. E. Ilgın, S. Göktaş

**Affiliations:** 1 Psychiatry, Marmara University Pendik Research and Education Hospital; 2 Psychology, Yeditepe University, İstanbul, Türkiye

## Abstract

**Introduction:**

Kleptomania is an impulse control disorder characterized by the inability to resist recurrent urges to steal objects that are not needed for personal use or have little monetary value. Some individuals may exhibit only a limited number of theft behaviors. Those diagnosed with this condition may face legal proceedings as a result of actions carried out due to their inability to suppress these impulses. In this report, we present the psychiatric assessment findings of a case with a history of two theft incidents.

**Objectives:**

This case report evaluates the clinical and psychometric profile of a male patient exhibiting kleptomania-like behavior within a psychiatric context. Data were obtained through structured and semi-structured clinical interviews, as well as psychometric tests including the Beck Depression Inventory (BDI), Beck Anxiety Inventory (BAI), Yale-Brown Obsessive Compulsive Scale (Y-BOCS), and Barratt Impulsiveness Scale-11 (BIS-11).

**Methods:**

Sociodemographic and medical history data were obtained through structured clinical interviews. Behavioral information regarding the theft incidents, including planning and subjective experience, was collected. Psychometric assessments included the BDI, BAI, Y-BOCS, and BIS-11 to evaluate mood, anxiety, obsessive-compulsive symptoms, and impulsivity.

**Results:**

K.C., born in 1974, divorced, high school graduate, lives in Istanbul. Formerly employed in the textile business with an income approximately two to three times the minimum wage, he declared bankruptcy in 2017 and divorced in 2019. Post-divorce, he experienced aggression, remorse, social withdrawal, and loneliness, which improved after reconciliation with his spouse. He has OCD, previously treated with sertraline (100 mg/day) and risperidone (0.5 mg/day), with noted benefit. Currently, he earns around the minimum wage, and his sleep and appetite are within normal limits. He also has chronic thyroid disease, treated with levothyroxine (75 μg/day). In 2024, he committed theft twice within one hour. He reported no planning, describing the acts as impulsive and attention lapses. He later emphasized his intention to avoid engaging in such acts again. Following the second incident, he was apprehended. Court proceedings are ongoing, but he compensated the complainant, who withdrew charges.

**Conclusions:**

Most patients with kleptomania do not seek psychiatric support voluntarily, and those who are apprehended during theft become involved in the forensic process, making it difficult to reach individuals in this group. This case illustrates that kleptomania-like behaviors may be frequently encountered in forensic settings and that diagnostic uncertainties are clinically significant. The fact that the patient engaged in theft behavior only twice and explained these acts as “forgetfulness” and “momentary impulsivity” provides limited evidence for diagnostic purposes.

**Disclosure of Interest:**

None Declared

